# ^19^F NMR studies provide insights into lipid membrane interactions of listeriolysin O, a pore forming toxin from *Listeria monocytogenes*

**DOI:** 10.1038/s41598-018-24692-6

**Published:** 2018-05-02

**Authors:** Mirijam Kozorog, Marc-Antoine Sani, Martina Lenarčič Živković, Gregor Ilc, Vesna Hodnik, Frances Separovic, Janez Plavec, Gregor Anderluh

**Affiliations:** 1 Department of Molecular Biology and Nanobiotechnology, National Institute of Chemistry, Hajdrihova 19, 1000 Ljubljana, Slovenia; 2 Graduate School of Biomedicine, Medical faculty, University of Ljubljana, 1000 Ljubljana, Slovenia; 3 School of Chemistry, Bio21 Institute, The University of Melbourne, Melbourne, VIC 3010 Australia; 4 Slovenian NMR Centre, National Institute of Chemistry, Hajdrihova 19, 1000 Ljubljana, Slovenia; 5 EN-FIST Centre of Excellence, Trg Osvobodilne fronte 13, 1001 Ljubljana, Slovenia; 6 Faculty of Chemistry and Chemical Technology, University of Ljubljana, Večna pot 113, 1000 Ljubljana, Slovenia

## Abstract

*Listeria monocytogenes* is a mammalian pathogen that causes gastroenteritis, miscarriages and infections of the central nervous system in immunocompromised individuals. Its main virulence factor is listeriolysin O (LLO), a pore-forming cholesterol-dependent cytolysin (CDC), which enables bacterial escape from the phagolysosome and contributes to bacterial pathogenicity. Details of cholesterol (Chol) recognition and membrane binding mechanisms by LLO are still not known. Here we used ^19^F-NMR spectroscopy in order to assess LLO-Chol interactions in solution and in a Chol-rich membrane environment. LLO has six tryptophan residues located in the region of the molecule that is first in contact with lipid membranes. ^19^F-LLO, which contained 5-fluoro-tryptophans, was prepared by using isotopic labelling in an *E. coli* expression system. Signals in the ^19^F-NMR spectrum of ^19^F-LLO were unambiguously assigned by using a series of single Trp → Phe point mutations. The results employing various cholesterol preparations in solution indicate that tryptophan residues are not directly involved in Chol binding in solution. However, significant chemical shift changes were observed upon LLO binding to Chol-rich membranes, highlighting the role of tryptophan residues in membrane interactions (W512) and oligomerisation (W189 and W489).

## Introduction

*Listeria monocytogenes* is an intracellular pathogenic bacterium, which is a causative agent of the food-borne disease listeriosis. *L. monocytogenes* has a complex life cycle that includes entering the cells by phagocytosis, escape from the phagocytic vacuole into the cytosol, replication and spreading to other cells and through tissues. Listeria uses a “tool kit” of virulence factors that promote these different steps^1–3^. The most important virulence factor is listeriolysin O (LLO), a pore-forming toxin with various roles in listeria pathogenesis^4^. LLO is shown to be important for entry of listeria in cells^5^, as well as for escape of bacteria from phagocytic vesicles in the cytosol of the cells^6^. This is a crucial step since it allows bacterial escape from a hostile environment of phagocytic vesicles into the cytosol, where bacteria can replicate and then spread to other cells. As a pore-forming protein, the role of LLO is to form pores in phagocytic membranes, which subsequently lead to the destruction of phagosomal membranes and transfer of bacteria to the cytosol^6–8^.

**Figure 1:**
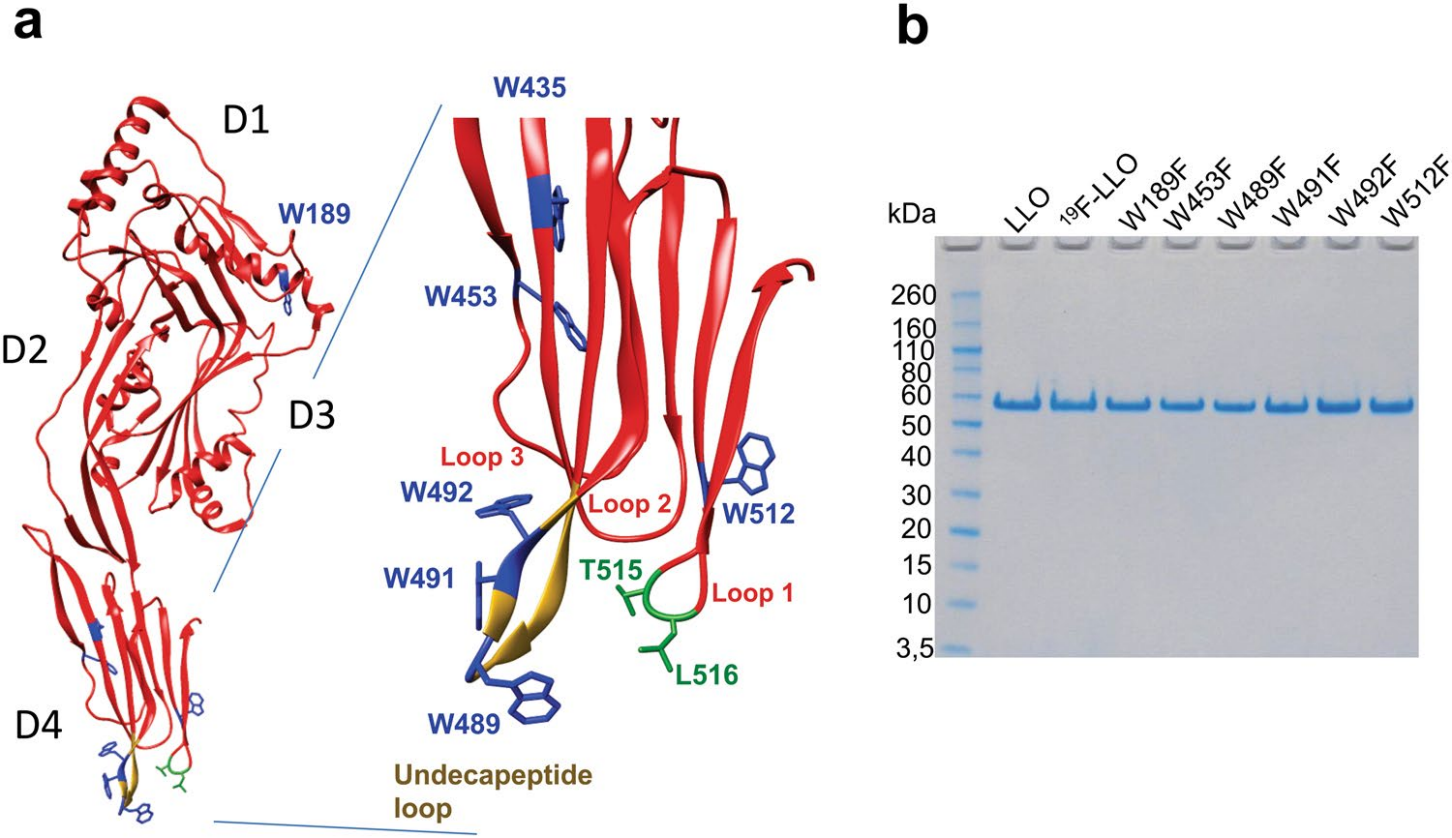
Structural model of LLO and purified LLO protein variants. (**a**) Structural model of LLO (PDB 4CDB)^16^. Domains 1–4 are designated D1–D4. Trp residues are designated in blue. The Chol-recognition motif in D4 is denoted in green. The inset shows enlarged D4, where relevant structural parts are labelled, and (**b**) SDS-PAGE analysis of purified LLO, ^19^F-LLO and mutants employed in the study.

LLO belongs to the cholesterol-dependent cytolysins (CDCs), a large family of bacterial pore-forming toxins (PFTs) found predominately in Gram-positive bacteria^9,10^. CDCs possibly represent the best studied family of PFTs, for which is known in great detail the molecular mechanisms that lead to formation of transmembrane pores from the assembly of soluble monomeric proteins once they are bound at the surface of lipid membranes^10^. Several structures of CDCs have been determined^11–15^, including that of LLO^16^. CDCs are characterised by organisation of the molecule into four domains, where each domain has a particular role in the pore-forming process. Domain 4 (D4) provides the first contact of the protein with the lipid membrane. D4 is folded as a β-sandwich that has, at the bottom of the molecule, three structural loops (Loop 1–3) and a tryptophan-rich undecapeptide, which is conserved among all CDCs (Fig. 1). D2 links D4 with D1 and provides the required flexibility. D3 is linked to D1 and possesses two clusters of α-helices that rearrange during the pore-forming process to form β-hairpins. Each monomer thus contributes two β-hairpins in a final transmembrane β-barrel assembly. These details are known to a large extent from the structural work and extensive mutagenesis studies of perfringolysin O (PFO), a CDC from the Gram-positive pathogenic bacterium *Clostridium perfringens*^9,10,17^, and pneumolysin O (PLY), a CDC from *Streptococcus pneumoniae*^18^.

Although LLO is very similar in sequence and structure to PFO and PLY, some notable differences exist. LLO is a pH-dependent cytolysin, which is crucial for its biological activity. Unique among CDCs, LLO possesses a so-called pH sensor, a triad of acidic amino acid residues in D3, that is responsible for the protein unfolding at temperatures above 30 °C and at physiological pH values of ~7.4^19,20^. This assures rapid inactivation of the toxin once Listeria is released from the phagolysosome into the cytosol. At pH values below 6.0, characteristic to phagolysosome compartments, LLO remains stable. Also LLO does not seem to form well-defined circular pores similar to those of PFO. Instead, LLO preferentially forms arcs, oligomeric assemblies lined on one side by a lipid membrane, which are conductive and function as pores^5,19,21–23^.

The exact mechanism of membrane binding and cholesterol (Chol) recognition is not entirely understood for the whole family of CDCs. We have shown previously that LLO requires a threshold of 30–40 mol % of Chol in lipid membranes for efficient binding and pore-formation^24^, which is analogous to other CDCs^25–28^. *In vitro* assays with soluble Chol preparations provide some quantitative information about interactions of Chol with CDCs. For example, *in vitro* pre-treatment of LLO with Chol in 1:15 ratio (LLO:Chol) resulted in 200-fold decreased activity of LLO^29^; and PLY was shown to bind to Chol in roughly 1:1 stoichiometry^30^. Fluorescence data also indicate that Chol interacts with PLY water-exposed tryptophan (Trp) residues that are positioned in the membrane facing part of D4^30^. However, molecular details of Chol recognition by LLO (or CDCs) are not known. Surface plasmon resonance (SPR) analysis of LLO binding to membranes that contained different sterols showed that binding was not affected when Chol analogues with modifications in the ring system or acyl chain were used but was significantly impaired when analogues with changed 3β-hydroxy group were used, such as cholesteryl acetate or 5-cholesten-3-one^24^. This indicates that LLO-Chol recognition is mediated through the underside of D4 and the 3β-hydroxy group of Chol. Indeed, the conserved threonine-leucine (Thr-Leu) pair in Loop 1, that is positioned in close proximity to Trp-rich undecapeptide at the base of D4 (Fig. 1), was recently shown to be important for Chol recognition by PFO^31^ and PLY^13^. A requirement for a proper structural motif on a Chol molecule for binding is also underscored by the fact that PFO can bind to Chol but not to epiChol, an analogue with different orientation of the 3-hydroxyl group^31–33^.

In this paper, the interactions of LLO with Chol and lipid membranes using ^19^F NMR spectroscopy are reported. An LLO variant was prepared, which contained 5-fluoro-tryptophans (5F-Trps) instead of the Trp residues. We wished to obtain more insight into the LLO-membrane interactions by monitoring the chemical environment of the 5F-Trps in different membrane systems. Trp residues are an excellent probe for studying LLO-membrane interactions, since six out of seven Trps in LLO are located in D4, some of which are in the vicinity of the two residues important for Chol recognition (Fig. 1). ^19^F NMR spectroscopy has proven to be a sensitive and reliable method for studying the chemical environment of fluorine atoms introduced at specific locations in the target protein molecules^34–37^ and has found use in fragment-based drug discovery^38^. We previously used Trp labelling and ^19^F NMR to provide molecular details of lipid interactions for another pore-forming toxin, equinatoxin II from sea anemone^39^.

## Results and Discussion

### ^19^F labelled LLO and its Trp mutants bind to Chol-rich membranes

In this study we aimed to gain insight into cholesterol recognition and membrane binding by LLO. The LLO molecule has seven Trp residues in its primary structure. While W189 is located in D1, the other six are located in D4. Four of these are located at the bottom of D4 (Fig. 1a) and most likely participate in membrane binding and potentially could have effects on cholesterol recognition by LLO. Three of these (W489, W491 and W492) are located in the highly conserved Trp-rich undecapeptide loop. Additionally, W512 is positioned in spatial proximity to Thr-Leu pair in loop 1 that is important for Chol recognition in PFO^31^. To investigate the molecular role of Trp residues upon Chol binding by NMR we produced wild-type LLO where all seven Trp residues were ^19^F isotopically labelled (^19^F-LLO). A previously established expression system was used that employs a Trp auxotroph of *E. coli*^39^ and 5F-Trp was added to the growth medium as the only source of Trp. For ^19^F NMR peak assignment, ^19^F labelled Trp mutants of ^19^F-LLO were prepared, where individual Trp was successively replaced by a phenylalanine (Phe) residue. Six LLO Trp mutants, W189F, W453F, W489F, W491F, W492F, and W512F, were prepared. Along with LLO and ^19^F-LLO they were successfully expressed in *E. coli* and purified to homogeneity (Fig. 1b). Circular dichroism spectra showed no structural changes in Trp mutants compared to the ^19^F-LLO protein (Fig. S1). We were unable to produce W435F in sufficiently large quantities and, therefore, did not use it in our subsequent NMR experiments. The side chain of W435, in contrast to other Trp residues, is not exposed to the solvent, but rather packed in by the side chains of surrounding amino acids (Fig. 1a). Moreover, H_ε1_ of W435 in LLO is hydrogen-bonded to the side chain of E437. Trp to Phe substitution, therefore, most likely changed the folding and/or stability of the LLO, which precluded its production in large quantities.

**Figure 2:**
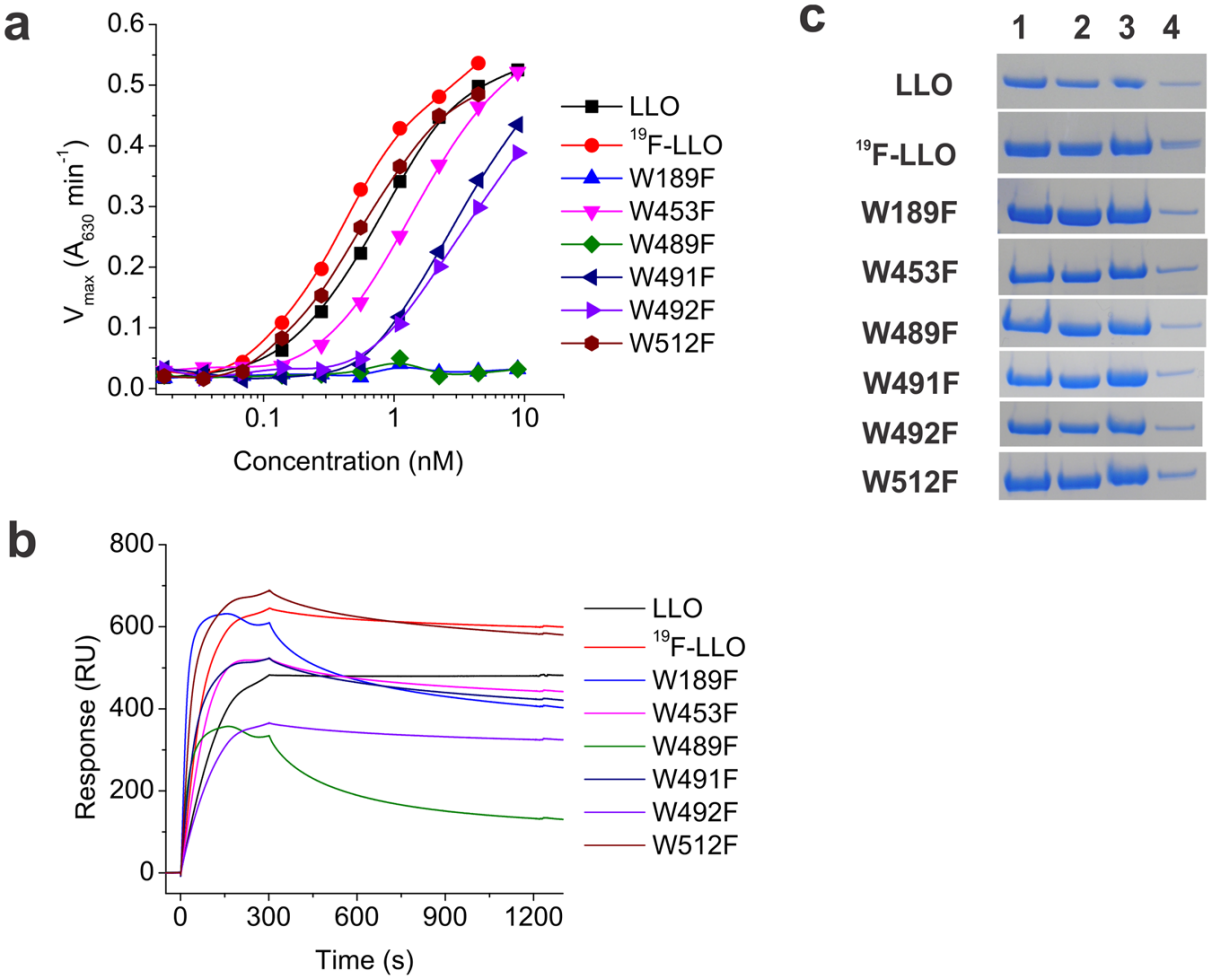
Hemolytic activity and membrane binding of LLO protein variants. (**a**) Hemolytic assay of LLO and mutants used in the study, (**b**) SPR analysis of LLO protein variants binding to lipid membranes composed of POPC:Chol (1:1), and (**c**) sedimentation assay, which indicates binding of proteins to multilamellar vesicles composed of POPC:Chol (1:1). (1) 2 μg of the protein; (2) supernatant after centrifugation of the protein samples incubated in the absence of multilamellar vesicles; (3) pellet and (4) supernatant after centrifugation of the protein samples incubated in the presence of multilamellar vesicles.

Various functional and membrane-binding assays were performed with the ^19^F residue-specific isotopically labelled proteins and compared to unlabelled LLO in order to check the functionality of the produced protein samples. The ^19^F-LLO showed similar activity and membrane-binding properties to LLO (Figs 2 and S2). Although hemolytic assays showed some differences, except for W189F and W489F, the mutants showed considerable permeabilising activity (Fig. 2a). Mutations of two other undecapeptide Trp residues (W491 and W492) also resulted in 4-fold lower hemolytic activity. The membrane-binding capacity of the mutants to 1-palmitoyl-2-oleoyl-*sn*-glycero-3-phosphocholine (POPC):Chol (1:1) liposomes was also checked. All protein variants exhibited similar membrane-binding in the SPR (Fig. 2b) or sedimentation assay using multilamellar vesicles (Fig. 2c, full gels in Fig. S2). These results suggest that substitution of Trp side chains with 5F-Trp residues did not influence the structure or activity of the LLO protein. Similarly, substitutions of individual Trp residues to Phe retain structural features of LLO, membrane-binding capacity and, with the exception of W189F and W489F, hemolytic activity.

### ^19^F NMR spectra of ^19^F-LLO in aqueous solution and assignment of peaks

**Figure 3:**
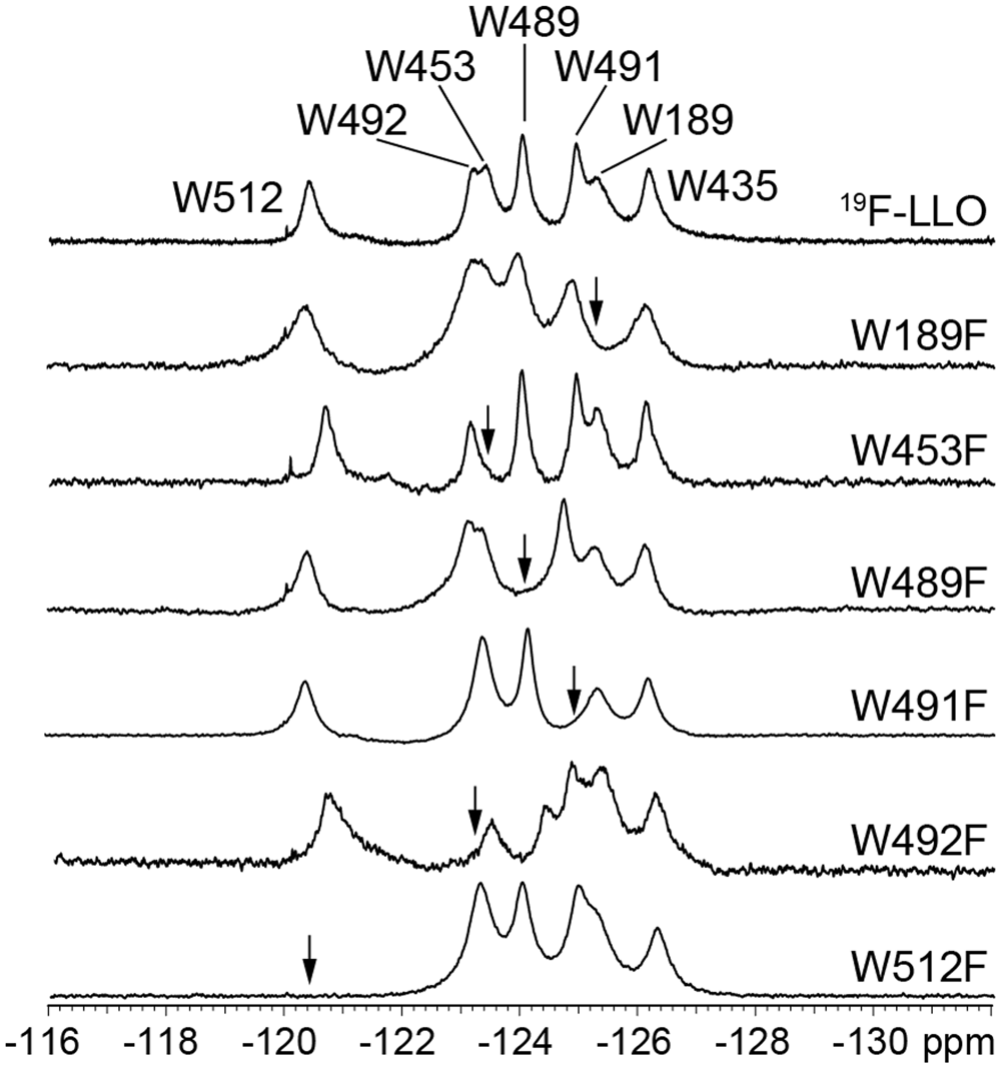
^19^F NMR spectra of ^19^F-LLO and its Trp mutants in MES buffer. The protein concentration was between 0.1 to 0.3 mM. Arrows represent the lack of signal of the Trp residue that was mutated to (isotopically unlabelled) Phe. Assignment of Trp residues is given by residue number in ^19^F-LLO spectrum.

The 1D ^19^F NMR spectra of ^19^F-LLO and Trp mutants were acquired. The ^19^F NMR spectrum of ^19^F-LLO in aqueous solution showed seven well-resolved peaks (Fig. 3). In comparison to ^19^F-LLO, ^19^F NMR spectra of single point Trp → Phe mutants exhibited six signals. With aid of the missing peak in individual ^19^F NMR spectra of mutants, all seven peaks were assigned to particular Trp residues (Fig. 3). The assignment was unambiguous (Table 1), although for some mutant samples we observed changes in the chemical shifts of the signals of the remaining six Trp residues. As expected, introduction of single tryptophan substitution of residues belonging to the conserved undecapeptide loop influenced the chemical shifts of other neighbouring Trp residues. In particular, W489F mutation caused downfield shifts of W491 and W492. Similarly, W491F substitution affected chemical shifts of W489 and W492, which both experienced subtle upfield shifts. In contrast, substitution of W492F caused upfield movement of W489 peak, while W491 moved downfield. Additionally, W492F substitution affected the chemical shift of W512 as well, which moved upfield. Chemical shift changes associated with Trp → Phe substitution of residues W489, W491 and W492 indicate small structural perturbations limited to the undecapeptide loop residues or cause only minor rearrangement of other residues (namely W512) without significantly altering the structure and/or activity of LLO protein. Substitution of W189 caused negligible chemical shift changes for other Trp residues, consistent with the position of W189 in D1 away from other six Trp residues in D4. W453F substitution affected the chemical shift of W512 which moved upfield, while W512F led to upfield chemical shift changes for residues W435 and W492. Mutual effect of residues W492 and W512 on each other may be allosteric by nature although, in general, these minor chemical shift changes for all residues reflect that the fold of LLO is preserved for all individual substitutions. Furthermore, due to minimal chemical shift changes, the assignment of W435 was easily done by elimination and unequivocally assigned to the peak at δ of −126.2 ppm.

**Table 1:** ^19^F NMR chemical shifts of Trp residues.

Residue	δ (ppm)	Linewidth at half height (ppm)
W189	−125.2	0.3
W435	−126.2	0.3
W453	−123.4	0.3
W489	−124.0	0.3
W491	−124.9	0.2
W492	−123.2	0.5
W512	−120.4	0.3

### LLO interaction with various Chol preparations

**Figure 4:**
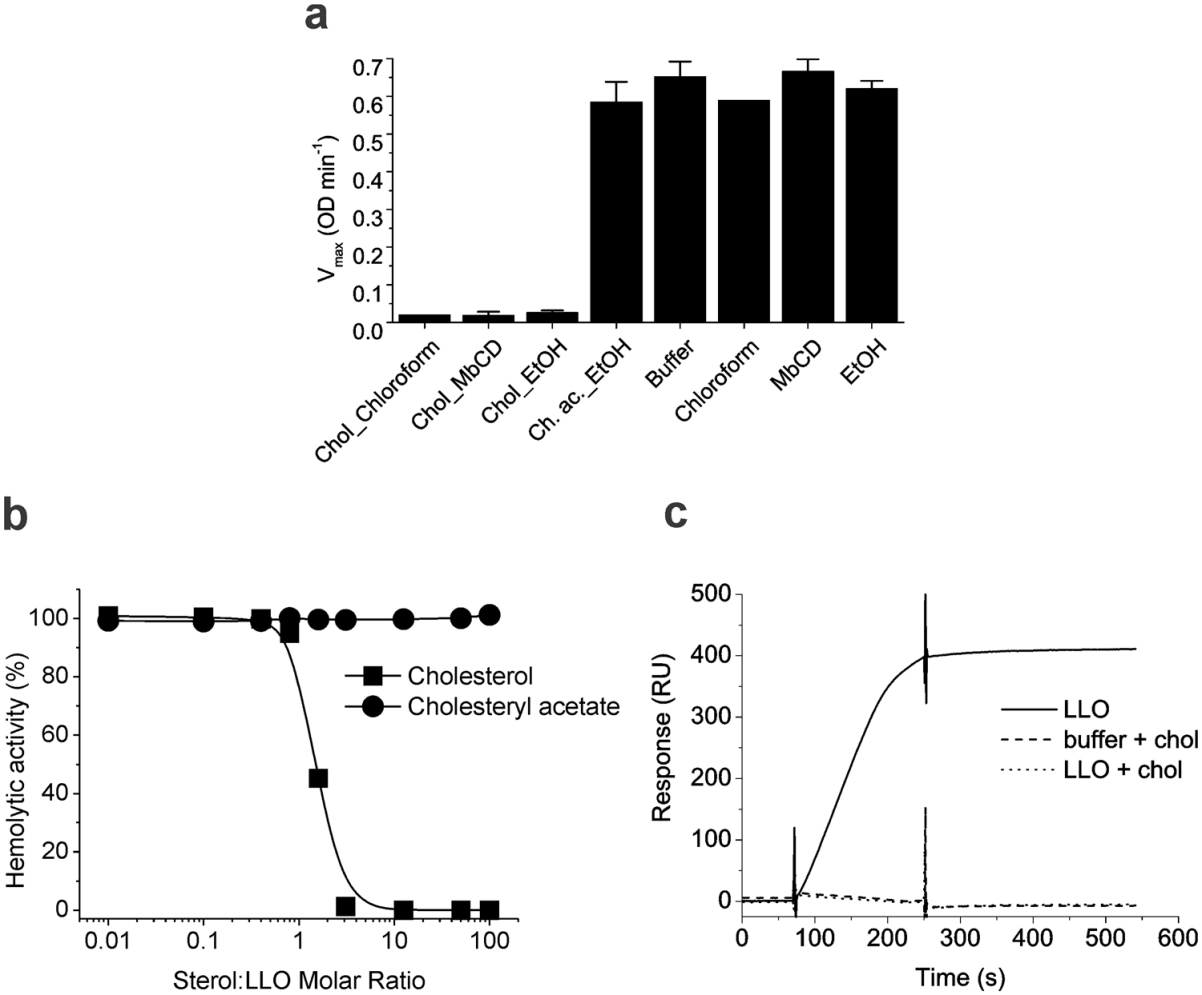
Interaction of LLO with various Chol preparations in solution. (**a**) Hemolysis maximal rate (OD min^−1^) of 22 nM LLO in the presence of various solutions as indicated in the panel. n = 3, average ± S.D. EtOH, ethanol; Ch. ac., cholesteryl acetate; MbCD, MβCD. (**b**) Hemolytic activity of 3.6 nM LLO after 30 min incubation in the presence of different concentrations of Chol and cholesteryl acetate in ethanol solutions. n = 3, average ± S.D., and (**c**) SPR analysis of LLO binding to lipid membranes composed of POPC:Chol (6:4) lipid vesicles after 20 min incubation with Chol in ethanol at 10:1 molar ratio (Chol:LLO) compared to non-treated LLO and buffer with Chol.

To obtain more details on direct LLO-Chol interactions, we incubated LLO with Chol prepared in different solvents. Our data showed that LLO incubation with Chol, dissolved in organic solvents, chloroform or ethanol, or in a complex with MβCD, decreased hemolytic activity (Fig. 4a). On the other hand, organic solvents or MβCD itself, used at the same concentrations as in the assay in the presence of Chol, had no effect on LLO activity. This suggests that the decrease in haemolytic activity was only due to irreversible LLO-Chol interaction that reduces the amount of the functional protein able to form pores on erythrocyte membranes. The Chol:LLO molar ratio that halved hemolysis maximal rate (V_max_) was determined to be 1.1 ± 0.5 (Fig. 4b), which correlates with 1:1 stoichiometry of sterol binding, similar to other CDCs, i.e. PLY^30^. In contrast, Chol analogue cholesteryl acetate with changed 3β-hydroxyl group did not affect LLO hemolytic activity at the molar ratios tested (Fig. 4b) in agreement with Bavdek *et al*.^24^. Furthermore, SPR results show that LLO, incubated with Chol in ethanol at 10:1 Chol:LLO molar ratio did not bind to Chol-rich vesicles, immobilised on the sensor chip (Fig. 4c). These results show that Chol in solution interacts with the membrane binding part of the LLO molecule and thus prevents its association with lipid membranes.

**Figure 5:**
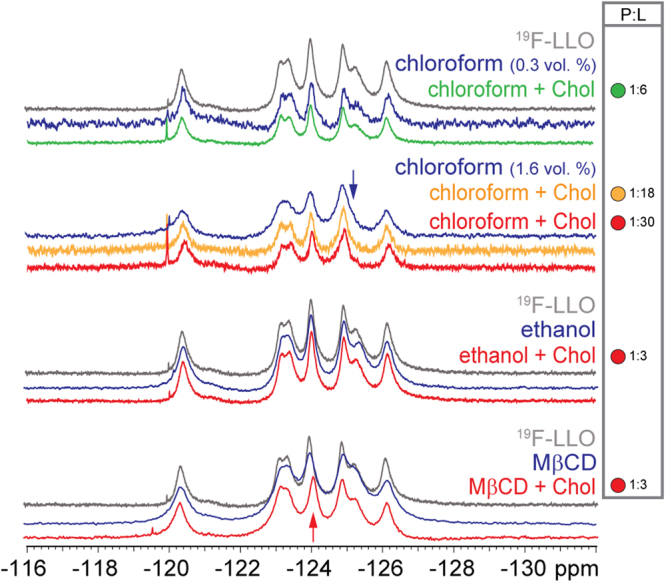
Interaction of LLO with Chol in solution as monitored by ^19^F NMR. ^19^F NMR spectra of 0.3 mM ^19^F-LLO treated with Chol dissolved in chloroform (1:6, 1:18 and 1:30 ^19^F-LLO:Chol molar ratios), ethanol or in a complex with MβCD (both 1:3 ^19^F-LLO:Chol molar ratio) compared to ^19^F-LLO spectra. P:L, protein:lipid (cholesterol) molar ratio.

Potential interaction of Chol with LLO and influence of different solvents in the Chol preparations were additionally monitored with the use of 1D ^19^F NMR spectra. Chemical shift changes of ^19^F-LLO in the presence of Chol prepared in different solvents can provide information about the interaction of Trp residues in LLO binding to free Chol in solution and when positioned in lipid membranes. ^19^F NMR spectra show that there were no chemical shift changes with a couple of exceptions that are a consequence of solvent addition to the protein sample (Fig. 5). Addition of 1% (v/v) of chloroform affected the position of W189 signal which moved downfield and overlapped with W491 (labelled with a blue arrow in Fig. 5). No additional changes of chemical shifts were observed in the presence of Chol in the LLO-chloroform sample. Ethanol alone or in addition with Chol in the protein sample did not perturb any of the Trp resonances. In comparison to the ^19^F-LLO spectra, addition of Chol in MβCD caused a small upfield chemical shift change of W489 (labelled with a red arrow in Fig. 5). The same amount of MβCD in buffer without the Chol did not cause any changes in the ^19^F-LLO spectra, suggesting W489 shift change is caused by the interaction of LLO with Chol-MβCD complex. Incorporation of Chol into the MβCD complex could create a membrane-like environment and facilitate ^19^F-LLO binding and W489 interaction either with Chol itself or with the Chol-MβCD complex. Although hemolytic assays and SPR indirectly confirm interaction between LLO and free Chol in solution, ^19^F NMR spectra in solution indicate that Trp residues were not involved in the interactions due to lack of any significant perturbations for most of the Trp resonances.

### Solid-state ^19^F NMR spectra of membrane-bound LLO and assignment of peaks

**Figure 6:**
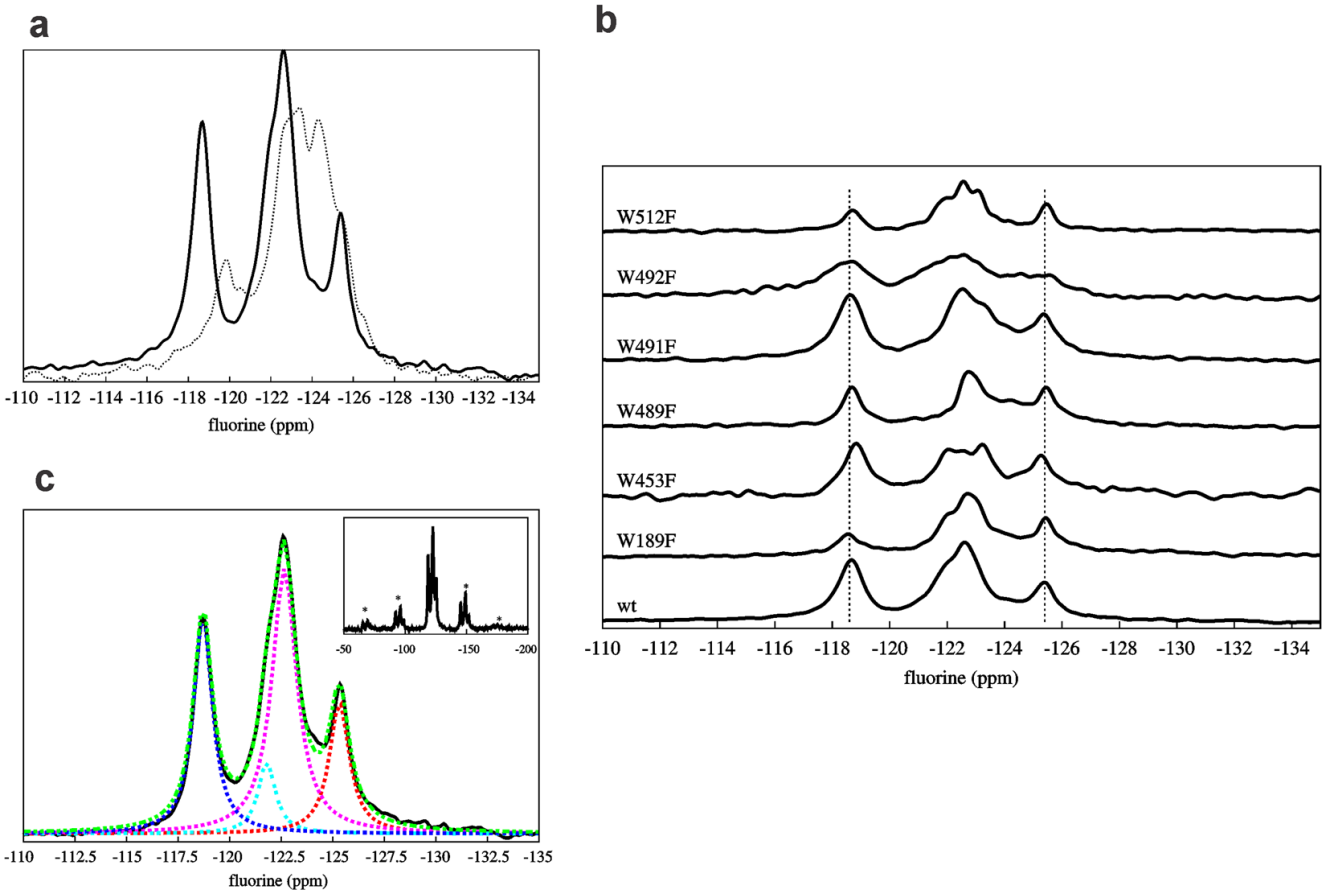
Comparison of solid-state NMR spectra of ^19^F-LLO protein when bound to POPC/Chol vesicles and free in MES buffer with Trp peaks assignment. (**a**) Solid-state ^19^F spectra of free LLO in MES buffer (dotted line) and when bound to multilamellar vesicles composed of POPC/Chol (solid line), (**b**) Assignments of peaks with single point Trp mutants. The fits to peaks are reported in Table 2, and (**c**) Fitting of Lorentzian functions to ^19^F NMR MAS spectrum of the ^19^F-LLO bound to POPC/Chol membranes (see Table 2). Experimental data - black, overall fit - green, individual components - red, magenta, cyan, blue. Inset shows spinning side bands in the MAS solid-state ^19^F-LLO NMR spectra.

POPC/Chol (1:1) multilamellar vesicles were formed with ^19^F-LLO bound in order to study the environment of 5F-Trp residues in membrane-bound LLO. The ^19^F solid-state NMR spectrum of bound protein was compared to that of the ^19^F-LLO protein in MES buffer (Fig. 6a), recorded without spinning. Although the solid-state NMR spectrum of the protein alone (Fig. 6a) shows less resolution than the solution NMR spectrum of the same protein (Fig. 3) the chemical shift positions could be mapped to those in solution. For example, the absence of the middle peak at δ −124.0 ppm, assigned to W489, is observed also in solid-state spectrum at δ −123 ppm. Trp residues that experience significant changes in the environment upon membrane interactions would be expected to experience a change in ^19^F chemical shift in the presence of POPC/Chol lipid vesicles, e.g. Trp residues involved in membrane binding or those that are located in protomer-protomer interfaces. Indeed, several notable changes were observed in the solid-state magic angle spinning (MAS) NMR spectra of the membrane-bound ^19^F-LLO in comparison to the protein in solution (Fig. 6a).

Trp → Phe LLO mutants were incubated with lipid membranes and MAS ^19^F solid-state NMR spectra were recorded in order to confirm or re-assign the Trp chemical shifts. Figure 6b shows significant intensity reduction in the ^19^F spectra of bound LLO mutants compared to that of the ^19^F-LLO spectrum, which was used for assigning the seven ^19^F labelled Trp residues. ^19^F spectra of the ^19^F-LLO bound to membranes has three peaks that could be assigned to four groups of Trp residues (Fig. 6c, Table 2). Interestingly, some Trp residues underwent a significant change in chemical shift upon binding to lipid vesicles, in particular W512 and W189, while others, such as W435, were mainly unperturbed in comparison to the vesicle-free ^19^F-LLO spectrum. W189 experienced a 7 ppm upfield shift to δ 118.6 ppm in comparison to free LLO in MES buffer (Fig. 6a,b, Table 2). W189F binding to Chol-rich vesicles is not affected as seen in Fig. 2, but its hemolytic activity is decreased (Fig. 2a) and significant change in the chemical shift would mean it experiences differences in chemical environment upon binding, perhaps being involved in oligomerization or other pore-forming event. W189 is positioned in α4 helix in D1, where mutations of several residues were shown to have an effect on hemolytic activity; namely K175E and S176W mutants with mutations in the same α helix were not able to oligomerize into ring-shaped pores and that completely abolished LLO activity^16^. The loss of activity of W189F could therefore be due to its importance in oligomer formation. W512F mutant on the other hand exhibits undisturbed hemolytic activity (Fig. 2a), but changes in W512 chemical shift indicate the environment change upon Chol-rich vesicle binding, suggesting it might be inserted in the membrane. W512 is located in Loop 1 near the Thr-Leu pair, which was shown previously to contribute to Chol-rich membrane binding in PFO^31^ and, therefore, possible that W512 inserts in the membrane when LLO molecule is in bound and oligomerized state in the membrane. Further, W489 experienced a change in the chemical shift from (around) δ 124.0 ppm to δ 121.8 ppm. Together with the decreased haemolytic activity (Fig. 2a), it can be concluded that W489 is also involved in the pore-forming process, probably in the initial oligomerisation, since it is less than 4 Å from L461 in the neighbouring molecule of the oligomer rows of LLO crystals^16^. Recent cryo-EM structure of the membrane pore of PLY showed that, the LLO W489 analogue, W433 was involved in interaction with D4 of the neighbouring monomer^18^. Since LLO W489 is positioned the same as PLY W433 at the tip of D4 in the conserved Trp-rich undecapeptide, the two could have a similar role in oligomerization of monomers on the Chol-rich membrane. Spinning side bands in the MAS solid-state NMR spectra were also visible (Fig. 6c, inset), which indicate a significant reduction in tryptophan dynamics, as expected for a membrane-bound state.

**Table 2:** ^19^F solid-state NMR chemical shifts of Trp residues of the ^19^F-LLO bound to POPC/Chol membranes.

Tryptophan	Chemical shift (ppm)	Linewidth at half height (ppm)	Relative Intensity (a.u.)
W435	−125.4	1.0	5.0
W491, W492, W453	−122.6	1.4	14.7
W489	−121.8	1.0	2.8
W512, W189	−118.6	1.1	9.3

## Conclusions

Using ^19^F labelled Trp residues, the LLO regions involved in cholesterol and membrane interactions were monitored by comparison of solution and MAS solid-state ^19^F NMR. Significant chemical shift changes were observed for several Trp residues upon LLO binding to Chol-rich membranes when compared to the free LLO protein in solution. The results showed a change in chemical shift for W189, which is located in D1 of LLO. Since its hemolytic activity was decreased when mutated to Phe, but Chol-rich vesicles binding was unchanged, W189 is probably involved in one of pore-formation events, possibly interactions between monomers in the pore. Other tryptophans from D4 also exhibited changes in their chemical shifts (W512 in Loop 1 and W489 in the Trp-rich conserved undecapeptide), which indicates that they participate in LLO-membrane interactions. Although the hemolytic activity of W512F was not affected, the ^19^F solid-state NMR spectra of LLO mutants suggest that LLO binding to Chol-rich membranes involves W512, which is in proximity to T515 and L514 in Loop 1, both previously shown to be essential for Chol binding by another CDC member, PFO^31^. The hemolytic activity of LLO W489F, however, was strongly decreased. Also the change in the ^19^F chemical shift observed in the solid-state NMR spectra in ^19^F-LLO confirmed the importance of W489 in pore-forming events, most probably being involved in oligomerization through L461 of neighbouring monomers, which are in close proximity in LLO crystals^16^. However, no major changes were observed by solution NMR of ^19^F-LLO when associated with Chol in solutions, except minimal chemical shift change for W489 when Chol was in a complex with MβCD, indicating that the Trp residues are not directly involved in Chol recognition, but rather participate in membrane binding and other steps in pore-forming process.

## Materials and Methods

### Materials

All materials in this study were obtained from Sigma-Aldrich, USA unless stated otherwise. POPC was purchased from Avanti Polar Lipids (Alabaster, USA) and used without further purification.

### Cloning and preparation of LLO WT and LLO mutants constructs

Cloning and expression of the recombinant wild-type LLO was performed as previously described^22^. The LLO gene lacking the signal sequence (residues 25–529) was inserted into pPROExHtb vector after the hexa-histidine tag. The construct also contained a Tobacco Etch Virus (TEV) protease cleavage site between the histidine tag and the N-terminal side of the protein. Single point Trp mutations W189F, W435F, W453F, W489F, W491F, W492F and W512F were introduced into the LLO gene by using site-directed mutagenesis with single oligonucleotide as described in Shenoy and Visweswariah^40^. All constructs were verified by nucleotide sequencing (Eurofins Genomics).

### Expression and purification of LLO

The expressions of unlabelled LLO was carried out as described in Podobnik *et al*.^22^. The recombinant protein was expressed in *E. coli* BL21(DE3)pLysS strain. Cells were grown with shaking at 37 °C in 50 ml of Terrific Broth (TB) medium with 100 μg/ml ampicillin and 25 μg/ml chloramphenicol. When the OD_600 nm_ reached 0.9, 40 ml of the culture was used to inoculate 4 L of the TB medium. At an OD_600 nm_ of 0.7, expression of LLO was induced with 500 μM isopropyl β-D-1-thiogalactopyranoside and the temperature was lowered to 20 °C. The growth was stopped after 20 h by centrifugation at 6 000 *g*. The cells were frozen and stored at −20 °C. When thawed, the cells were suspended in 120 ml of the lysis buffer (50 mM BisTris (pH 6.5), 250 mM NaCl, 10% glycerol, 2 mM dithiothreitol (DTT), 1 mM benzamidine, 0.5 mg/ml lysozyme and 2 mM phenylmethylsulfonyl fluoride) and lysed by sonication. Lysed cells were centrifuged at 17 000 *g* and the supernatant was filtered through 0.22 μm polyvinylidene fluoride syringe filter unit (Millex). The filtrate was applied to the IMAC Sepharose HP column (GE Healthcare) with bound Ni^2+^ ions. The column was washed with 50 mM NaH_2_PO_4_ (pH 6.5), 300 mM NaCl and 5% glycerol, followed by step gradients of 100 and 240 mM imidazole. The histidine-tagged recombinant LLO (hisLLO) was eluted with 450 mM imidazole and was dialyzed overnight in the presence of TEV protease, prepared in-house. Cleaved hexa-histidine tag residues and TEV protease, which also contained histidine tag, were removed with another IMAC step and the LLO containing non-bound fraction was collected. The protein was concentrated and exchanged to the final MES buffer containing 20 mM MES, pH 5.7, 150 mM NaCl, 5% glycerol, 2 mM DTT.

### Production of ^19^F-LLO and ^19^F-LLO mutants

The genes for the wild-type LLO and its Trp mutants in pPROExHtb vector were transformed into Trp auxotroph strain *E. coli* BL21(DE3) trp::Tn10, which bears tetracycline resistance and grows only when Trp is present in the medium. Strain preparation and growth conditions are described previously^39^ and only minor modifications were made. Bacteria were grown with shaking at 37 °C in Lysogeny broth (LB) medium supplemented with 100 μg/ml ampicillin and 15 μg/ml tetracycline until they reached mid-exponential phase (OD_600 nm_ approximately 1.5). 10 ml of culture was transferred to 0.5 l LB medium supplemented with M9 salts and antibiotics, then grown with shaking at 20 °C to reach mid-exponential phase, followed by sterile centrifugation at room temperature. The pellet was washed with M9 salts and after second centrifugation transferred to M9 minimal medium with antibiotics, 2% (w/v) glucose, 1 mg/ml of biotin, 1 mg/ml of thiamine, and 1% Casamino acids that lack Trp. After one hour shaking at 20 °C final 0.4 mM 5F-Trp was added and the protein expression was induced with the addition of final 0.4 mM IPTG 30 min later. After 4 h the growth was stopped with centrifugation and one additional M9 salts pellet wash and the mutant proteins were purified as described above for the unlabelled LLO. All produced proteins were checked for purity on 4–12% Bis-Tris SDS-PAGE gel (Life Technologies) after mixing with SDS Sample Buffer (Life technologies) and 10 min denaturation at 70 °C. The gel image was not processed.

### Membrane-binding assay

Multilamellar vesicles (MLVs) were formed from POPC and Chol (1:1 mol:mol). Lipids were dissolved in chloroform and the solvent was evaporated using rotary evaporator. 10 mM MES buffer (pH 5.7) with 150 mM NaCl and 1 mM EDTA was added to dried lipid film which was freeze-thawed three times using liquid nitrogen to form 22 mM MLVs. Proteins (2 µg) were added to MLVs to give a final 1:2500 molar ratio (LLO:lipids). Samples were incubated 30 min at room temperature and then centrifuged for 15 min at 16 100 *g*. Clear supernatants were collected and pellets were washed once in MES buffer. All samples were then mixed with SDS Sample Buffer (Life Technologies) and denatured for 10 min at 70 °C. Total sample volumes were analysed for protein presence on 4–12% Bis-Tris gels (Life Technologies). As a control, each of the proteins was incubated in MES buffer without MLVs and treated the same. 2 µg of each protein was applied to gels as a control. The samples, derived from the same experiment were applied on 4 separate gels that were processed in parallel. Novex Sharp Unstained Protein Standard was applied twice on each gel and bands (all at 56.4 kDa) were cropped from original gels for final presentations. The gel images were not further processed.

### Surface plasmon resonance

The SPR measurements of LLO, ^19^F-LLO and LLO Trp mutants binding to vesicles were carried out on Biacore T100 apparatus (GE Healthcare) at 25 °C. Series S sensor chip L1 (GE Healthcare) was equilibrated in running buffer (10 mM MES, 150 mM NaCl, 1 mM EDTA, pH 5.7). Large unilamellar vesicles (LUVs) of POPC/Chol (1:1 mol:mol) were prepared by extrusion through 100 nm pores from MLVs, prepared as in the membrane-binding assay. The liposome-coated chip surface was prepared as described before^24^. Two flow cells were firstly cleaned with 60 s injection of 40 mM octyl β-D-glucopyranoside at a flow rate of 30 µl/min. The liposomes were captured freshly for each protein injection at a flow rate 2 µl/min with an average response of deposited LUVs of 772 ± 29 RU. To cover the remaining surface, 0.1 mg/ml bovine serum albumin (BSA) was injected for 180 s over both flow cells and the surface was allowed to stabilise for 240 s prior to the protein injections. Proteins were injected at 50 nM concentration for 300 s at 30 µl/min and dissociation was monitored for additional 900 s. Vesicles were removed with 60 s pulse of 50 mM NaOH, 0.5% sodium dodecyl sulfate (SDS) with additional 60 s pulse of 40 mM octyl β-D-glucopyranoside at a flow rate 30 µl/min.

To determine LLO binding to Chol, assays were performed using a Biacore X100 apparatus (GE Healthcare). L1 chip was equilibrated in running buffer (20 mM MES, 150 mM NaCl, pH 5.7) and LUVs were captured at a flow rate of 5 µl/min, followed by injections of 0.1 M NaOH (30 µl/min, two times 60 s) and BSA (5 µl/min). The responses of deposited LUVs were 6646 RU for POPC in reference cell and 7319 RU for POPC/Chol (3:2) in sample cell. Final 200 nM LLO in MES buffer and 2 µM Chol in ethanol were mixed and incubated for 20 min. The chip was firstly injected with MES buffer with ethanol, then with 2 µM Chol in same buffer with ethanol, followed by LLO/Chol mixture and LLO with ethanol, each for 180 s. The surface was stabilized for 300 s after each sample and lipid injection and 60 s after NaOH and BSA. Ethanol was 5% (v/v) in all samples. Other conditions were as above.

### Hemolytic assay

Bovine red blood cells (RBCs) were washed in erythrocyte buffer (EB, 140 mM NaCl, 20 mM TRIS, pH 7.4) at mild centrifugation until the buffer remained clear. RBCs were diluted in EB to reach A_630_ ~ 0.5, as determined with the microplate reader Synergy MX (Biotek, USA). 100 μl serial dilutions of purified protein samples were prepared in 96-well clear microtiter plates and 100 μl of RBC suspension was added to each well. A_630_ was measured every 20 s for 20 min at 25 °C. V_max_ (OD min^−1^) for each protein concentration was determined with Gen5 software (Biotek, USA) using linear regression with 3 data points. V_max_ data points were plotted against protein concentration. Curves were fitted with logistic function by using Origin 8.1 (OriginLab, USA).

### Hemolytic assays for monitoring interaction of LLO with cholesterol preparations

When evaluating Chol binding to LLO by hemolytic assay, the experiments were performed as above but with minor modifications. An incubation time was added to enable efficient sterol binding to the LLO protein and, for clarity, A_630_ was checked only at the final point of hemolysis at the 20 min time point. Chol was dissolved in chloroform (50 mg/ml) or ethanol and cholesteryl acetate was dissolved in ethanol, both to 240 µM. 3 µl of Chol in chloroform and 5 µl of the sterol solutions in ethanol were separately added to final 100 µl of LLO (5 µg/ml) and incubated for 30 min. 2-fold dilutions in erythrocyte buffer were made and hemolytic activity was compared to non-treated LLO in buffer without and with same volumes of organic solvents without Chol or cholesteryl acetate. The mixture of Chol and MβCD was prepared similarly as described in Johnson *et al*.^41^. 1 mg Chol was dissolved in chloroform/methanol mixture (1:1 v/v) in a glass vial (at a final 12.9 mM concentration) and dried with a rotary evaporator. Same molar concentration of MβCD (2.8 mg in 200 μl MES buffer, pH 5.7) was added to the glass vial. The mixture was thoroughly vortexed and sonicated for 30 min in ultrasonic bath and incubated overnight at 37 °C on a rocking platform at 200 rpm. The next day, 10 μl of Chol-MβCD mixture or its 10 and 100-fold dilutions were added to the same volume of LLO (final 40 μg/ml), incubated for 30 min and tested for hemolytic activity after adding EB and making 2-fold dilutions as described above. 60% of Chol was incorporated into MβCD mixture (final concentration 8.2 mM, determined with Free Chol E (Wako Chemicals GmbH) enzymatic assay); the rest remained unsuspended on the glass vial wall. The mixture solution appeared clear. At least 3 separate experiments were done for each LLO:Chol sample.

For Chol:LLO stoichiometry determination, 28.8 µM Chol in ethanol was prepared. 2-fold serial dilutions were made and 5 µl of each dilution was incubated with 14.4 nM LLO in final 100 µl EB at 100:1 to 0.01:1 Chol:LLO molar ratio. After 30 min, 2-fold dilutions were made in EB and same volume of RBCs was added. Hemolytic activities of the samples were calculated after 20 min from the differences between starting and final A_630_ values, which were compared to final absorbance of the RBCs, treated with LLO, incubated in buffer with ethanol without Chol. The final LLO concentrations in RBC suspensions were 3.6 and 1.8 nM. Cholesteryl acetate was prepared in ethanol and incubated with LLO at the same ratios as Chol. Ethanol was 5% (v/v) in all samples. Three separate experiments were conducted.

### Solution NMR experiments

#### Sample preparation

0.3 mM ^19^F-LLO proteins in MES buffer, substituted with 10% D_2_O were placed in 5 mm OD Shigemi susceptibility-matched NMR tubes. Chol in chloroform, ethanol or dissolved in MβCD (prepared as described above) were added to the ^19^F-LLO in MES buffer in final 1:6, 1:18, 1:30 (chloroform) and 1:3 (ethanol, MβCD) LLO:Chol molar ratios. They were incubated for 30 min and transferred to Shigemi tubes. Same volume and molar ratio of chloroform, ethanol or MβCD solution, all without Chol, were also added to the labelled protein and incubated prior signal recording.

#### ^19^F solution NMR

^19^F solution NMR spectra were recorded at 565 MHz on DD2 600 MHz spectrometer (Agilent Technologies) equipped with One NMR probe. All spectra were recorded at 25 °C and referenced to CF_4_. The spectrum of ^19^F-LLO was recorded using 80 k acquisitions. Number of scans of other samples varied between 18 k and 80 k, depending on the protein concentration. Recycle time (acquisition plus delay) was 1.0 s. Data processing was performed with VNMRJ version 4.2 and MestReNova version 11.0.1.

### Solid-state NMR experiments

#### Sample preparation

^19^F-LLO in MES buffer, pH 5.7 was added to lyophilized lipid mixture at the lipid:protein molar ratio 125:1. After 30 min of incubation the mixture was freeze-thawed 3 times in liquid N_2_ and centrifuged for 10 min at 15 000 *g* and 20 °C. Pellet was weighed and packed in rotor by centrifugation technique. Similarly, ^19^F labelled LLO single point Trp mutants were incubated with POPC:Chol (3:2) lipid mixtures and ^19^F NMR spectra were recorded for Trp peak assignment. To obtain ^19^F NMR static solid-state spectra of the free ^19^F-LLO in solution, 0.3 mM ^19^F-LLO in MES buffer was added directly to rotor.

#### ^19^F solid-state NMR

The ^19^F NMR experiments were conducted at 25 °C on a Bruker 400 MHz NMR equipped with HFXY 4 mm MAS probe. The ^19^F static and magic angle spinning (MAS) experiments at 10 kHz spinning speed were performed at 376.5 MHz, using a *ca*. 78 kHz single pulse excitation with *ca*. 55 kHz SPINAL64 ^1^H decoupling, a 5 s recycle delay, a spectral width of 250 kHz, 8 k complex points acquisition zero-filled to 16 k points and line broadening ranging from 50 Hz to 100 Hz. Spectra were externally referenced using trifluoroacetic acid (−76.5 ppm) prior to each MAS experiment. Typically 20 k scans and 16 k scans were acquired for the static and MAS ^19^F NMR spectra.

## Electronic supplementary material

Supplementary information
