# Supplementary material for: 19F NMR studies provide insights into lipid membrane interactions of listeriolysin O, a pore forming toxin from Listeria monocytogenes
Source: Sci Rep. 2018 May 2;8:6894. doi: 10.1038/s41598-018-24692-6 (PMC5931962; doi:10.1038/s41598-018-24692-6)
Supplement: Supplementary file 1 — Supplementary information [file 41598_2018_24692_MOESM1_ESM.docx]

Supplementary Material for

**^19^F NMR studies provide insights into lipid membrane interactions of listeriolysin O, a pore forming toxin from *Listeria monocytogenes***

Mirijam Kozorog, Marc-Antoine Sani, Martina Lenarčič Živković, Gregor Ilc, Vesna Hodnik, Frances Separovic, Janez Plavec, Gregor Anderluh*

* Corresponding author. Email: [gregor.anderluh@ki.si](mailto:gregor.anderluh@ki.si)

**This file includes:**

Materials and Methods

Figs. S1 and S2

**Matherials and Methods**

**Circular dichroism spectroscopy**

Far ultraviolet circular dichroism (CD) spectra of ^19^F-LLO and its Trp mutants were measured on a Chirascan CD spectrometer (Applied Photophysics) in a quartz cuvette with a path length of 0.1 cm. Spectra were measured in a 190-280 nm wavelength range, step size was 0.5 nm and integration time was 0.5 s. 5 scans were averaged for each spectrum. Base-line spectra were subtracted from each spectrum using Chirascan software. Spectra were processed, smoothed and converted with the same software. The concentration of ^19^F-labelled proteins was determined with Bradford assay (Bio-Rad) with a comparison to unlabelled LLO with known concentration, determined from A_280_. The concentrations of the ^19^F-labelled proteins in CD experiments were 0.10 (^19^F-LLO, W489F, W512F), 0.11 (W453F), 0.12 (W491F, W492F) and 0.13 mg/ml (W189F).

**Figure S1**

**
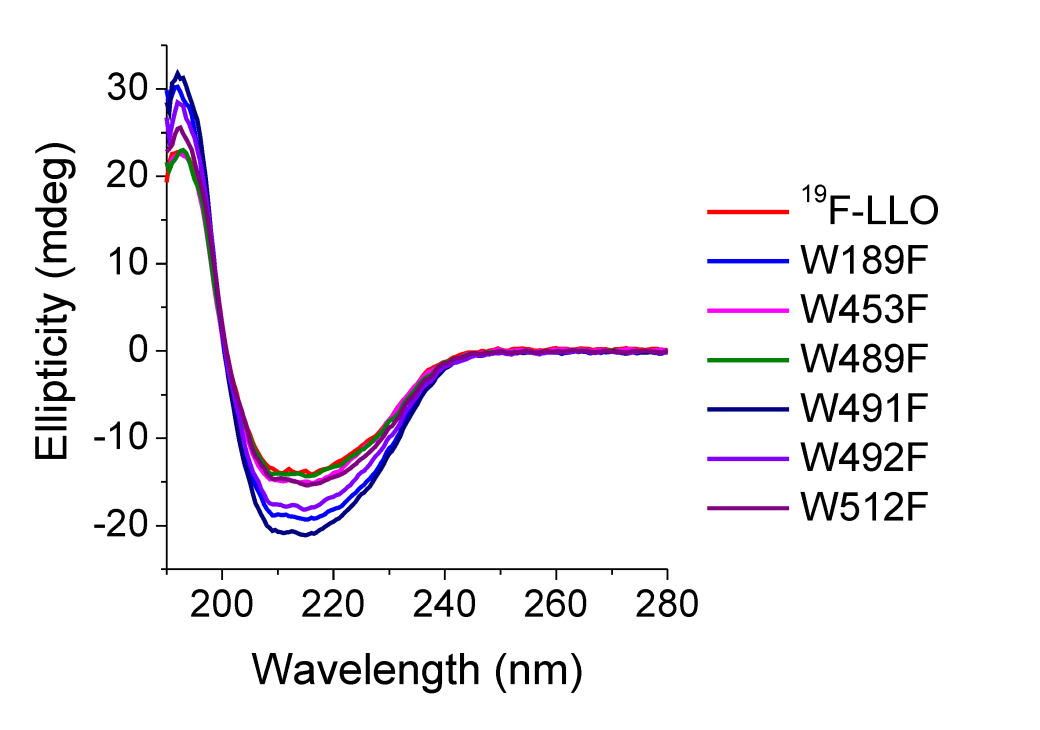
**

**Figure S1: Circular dichroism spectra of ^19^F-LLO and its Trp mutants used in the study.**

**Figure S2**


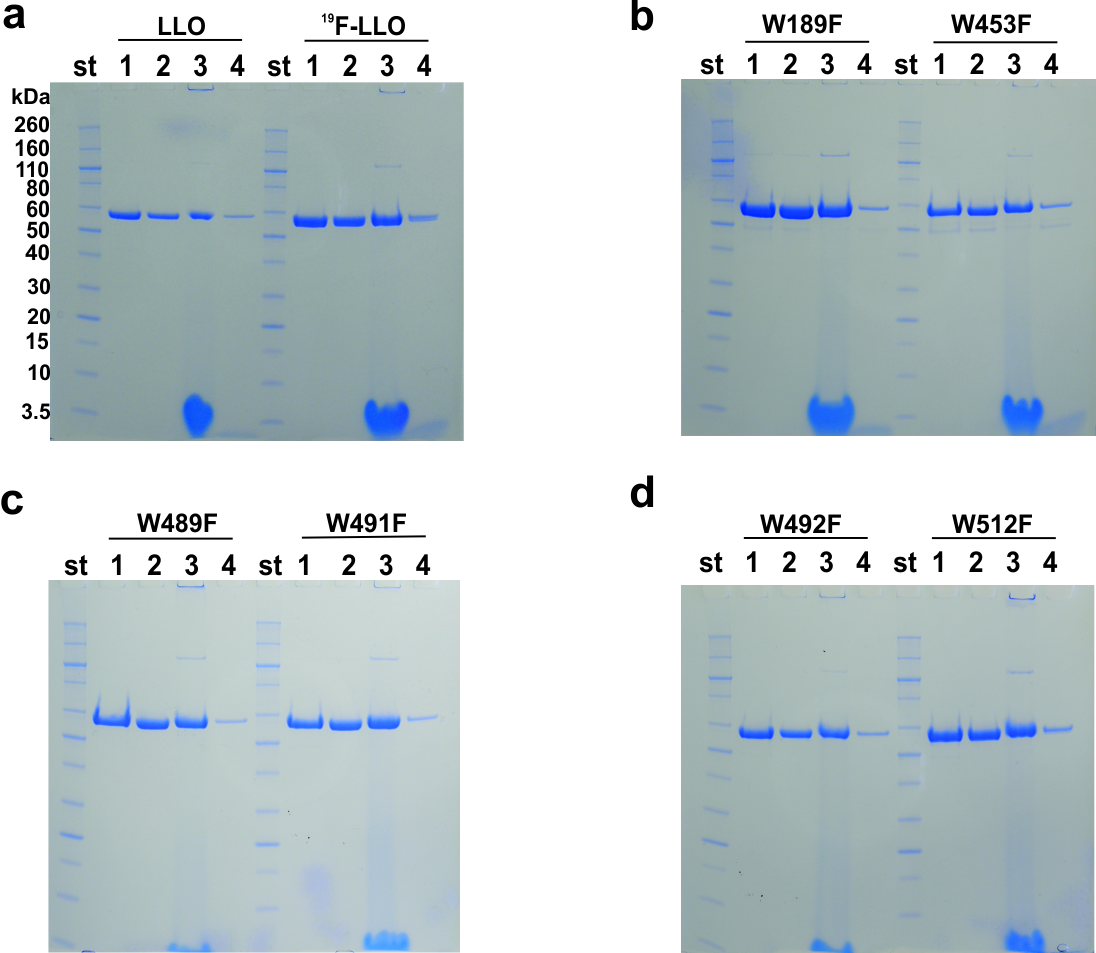


**Figure S2: SDS-PAGE gel images of sedimentation assay samples.** Two protein samples were loaded on each gel (**a**-**d**). All gels were run and processed in parallel. (1) 2 µg of the protein; (2) supernatant after centrifugation of the protein samples incubated in the absence of multilamellar vesicles; (3) pellet and (4) supernatant after centrifugation of the protein samples incubated in the presence of multilamellar vesicles. st, molecular weight standard. Protein bands are seen at 56,4 kDa position and bands at the bottom part of the gel represent lipids. Protein standards molecular weights, marked in **a** apply also for **b**-**d**.
